# Quackery

**Published:** 1877-05

**Authors:** 


### SELECTIONS.

### QUACKERY.

The following remarks by Dr. Felix Weise, D. D. S., at the close of his "Notes from a Dentist's Case-book," Published in the April No. of the "*British Journal of D. ntal Science*," are so appropriate and not by any means confined in their adaptation to England, that we cannot forbear giving them a place here. They will undoubtedly strike a few points in the United States.

He says:

I must now bring these notes to an end, and should like to avail myself of the opportunity to add a few words upon the relation which should subsist between the professional adviser and his patient, an alliance unfortunately frequently misunderstood. I am aware that the subject is one that should be approached with great delicacy, and that in the majority of instances the Dental surgeon has been selected by the patient for the well-earned fame and position his integrity and skill have gained for him. But unfortunately empiricism stalks so boldly before the public, our daily prints teem with so many tempting allurements, and our letter boxes groan under the surfeit of regularly supplied charlatan literature, that the poor patient cannot sometimes help being influenced by these means, and like the venturesome moth only learns, when too late, how little dependence can be placed on advertised ability and the loudly asserted integrity published in a public print.

This, however, is not the only evil, nor is it the greatest ; for if persons will be led astray by announcements that a moment's reflection should convince them are only delusive, they deserve to suffer. The great evil, and one that now permeates all classes of society, and I particularly wish to call attention to it, is the injury resulting from the professed teaching of these charlatan announcements. How painful to the well-informed and conscientious practitioner to be directed what he should do. To be told that such and such treatment, or this class of filling, or that style of mechanism, is suited to their individual wants. Patients will read these objectionable advertisements, and while reading think of their own cases. It is difficult to convince a too credulous public that every word in these puffing announcements is intended only to allure the unwary and that very nearly every statement put forward is untrue. That the *patent processes peculiar to the advertiser's manufactory* is a delusion, his special modes of operating only distiguished by a want of scientific knowledge. That he is himself as unprincipled as he is ignorant, and were he to cease putting forward his announcements his vaunted practice would fall to the ground. How would the physician act if dictated to as to the treatment he should adopt? The patient goes to him, not only with a belief in his skill, but also with faith in his integrity. To what purpose are our schools of instruction, our special hospitals, our regularly appointed lecturers, our stringent examinations, and our strict code of ethics, if the followers of these regulations are to be classed with the vulgar advertisers, the greedy adventurers? I appeal to every thinking individual, should such a state of things be encouraged? and the public do encourage these nefarious practices when they patronise a man who has to trumpet his own praise in the public papers, or indeed in any form that the heads of the profession disallow. I appeal to the medical faculty, ever ready to encouraged the members of an honorable profession, striving to free themselves from this incubus, and more than all I appeal to the press to discourage that which is dishonest, for if any book or advertisement states an untruth it is dishonest to promulgate the statement. If a certain section desire to make the profession a trade, let them do so, but act like honest tradesmen, do not say that you have special patents when you know that you have none, that you are original discoverers of some of the greatest improvements in science, or that your "exclusive system" offers advantage not to be obtained elsewhere. Do not openly advocate that which is most prejudicial to the health of your confiding patients, or have recourse to all kinds of subterfuge and falsehood unworthy of any description of trade.

It is such announcements as these that, alas, too frequently poison the minds of our patients and destroy their confidence. It is in opposition to such teaching I continue, as I have done before, to raise my voice, believing that the only true bond of union that holds the patient to his adviser, and the professional man to his patients is the mutual confidence in each other's integrity, and the firm belief that no solid advantages can accrue either on the one side or the other where that confidence is shaken.

